# Demodulation Method for Loran-C at Low SNR Based on Envelope Correlation–Phase Detection

**DOI:** 10.3390/s20164535

**Published:** 2020-08-13

**Authors:** Jiangbin Yuan, Wenhe Yan, Shifeng Li, Yu Hua

**Affiliations:** 1 National Time Service Center, Chinese Academy of Sciences, Xi’an 710600, China; ywh@ntsc.ac.cn (W.Y.); lishifeng@ntsc.ac.cn (S.L.); hy@ntsc.ac.cn (Y.H.); 2 School of Astronomy and Space Science, University of Chinese Academy of Sciences, Beijing 100049, China; 3 Key Laboratory of Precise Positioning and Timing Technology, Chinese Academy of Sciences, Xi’an 71060, China

**Keywords:** Loran-C, data demodulation, envelope correlation, skywave interference

## Abstract

Loran-C is the most important backup and supplement system for the global navigation satellite system (GNSS). However, existing Loran-C demodulation methods are easily affected by noise and skywave interference (SWI). Therefore, this article proposes a demodulation method based on Loran-C pulse envelope correlation–phase detection (EC–PD), in which EC has two implementation schemes, namely moving average-cross correlation and matched correlation, to reduce the effects of noise and SWI. The mathematical models of the EC, calculation of the signal-to-noise ratio (SNR) gain, and selection of the EC schemes are given. The simulation results show that compared with an existing method, the proposed method has clear advantages: (1) The demodulation SNR threshold under Gaussian channel is only −2 dB, a reduction of 12.5 dB; (2) The probability of the demodulated SNR threshold, being less than zero under the SWI environment, can reach 0.78, a 26-fold increase. The test results show that the average data availability of the proposed method is 3.3 times higher than that of the existing method. Thus, our demodulation method has higher engineering application value. This will improve the performance of the modern Loran-C system, making it a more reliable backup for the GNSS.

## 1. Introduction

The positioning, navigation, and timing (PNT) system is the key infrastructure in any country considering national economy and security. It provides PNT services for military, commercial, and civil users worldwide [1,2]. One of the high-precision ground-based PNT systems is Loran–C, which has advantages such as long-distance propagation, low frequency, high power, and goodstability [3,4,5,6]. These features make the Loran-C an ideal backup system for the global navigation satellite system (GNSS) in PNT applications [7,8,9,10,11], especially when GNSS signals are rejected or interfered. When using the signal transmitted from a Loran-C system to implement a timing function, the receiver must determine the time deviation (TD) between the local time and the standard time. The TD is composed of the time of arrival (TOA) and broadcast time (BT) of the signal. The TOA refers to the absolute propagation time of the current Loran-C pulse group signal from the Loran-C station to the current position of the receiver. The BT, which is obtained through data demodulation, refers to the time interval between the start time of the current Loran-C pulse group signal and the standard time. Currently, the international standard Loran-C signal system typically uses EUROFIX technology for data dissemination [12,13]. The EUROFIX datalink is implemented by an additional three-level pulse position modulation (PPM) of the Loran-C pulses. The above-mentioned Loran-C signal system is adopted in China’s BPL long wave time service system and Changhe 2 navigation system [14,15].

The signal-to-noise ratio (SNR) required for Loran-C data demodulation is higher than that for signal acquisition and detection. Therefore, the demodulation performance determines the timing capability of the Loran-C receiver. In recent years, studies on Loran-C signal receiving methods have mainly focused on ways of enhancing the accuracy of TOA measurements, such as by signal acquisition and detection [16,17,18], skywave identification [19,20,21,22], cycle identification [23,24,25], and additional secondary phase factor correction [26,27,28]. However, little attention has been paid to enhancing the demodulation performance of the PPM. The basic method used involves converting the PPM into phase modulation [14,29,30], i.e., the demodulation of the PPM is converted to the detection of the phase of the Loran-C pulse envelope. Based on the basic method, an envelope phase detection–majority decision (EPD–MD) method has been proposed in [14]. This method uses multiple phases of the orthogonal envelope to determine the modulation polarity via majority decision to improve the demodulation performance; however, the performance deteriorates sharply at low SNRs. In [29,30], a demodulation method based on signal matching correlation–pulse position detection (SMC–PPD) has been proposed. Since the matching correlation peak of the Loran-C pulse signal is not sharp, the SMC–PPD method cannot significantly improve the SNR performance. In addition, when there is skywave interference (SWI) in the received signal, the SMC–PPD method cannot detect the position of the Loran–C pulse signal correctly.

Therefore, this article proposes an envelope correlation–phase detection (EC–PD) method to demodulate the Loran-C signal at low SNRs. In this method, two EC schemes, namely moving average-cross correlation (MA–CC) and matched correlation (MC), are used to reduce the effects of noise and SWI, thus significantly improving the timing capability and sensitivity of the Loran-C receiver.

## 2. Materials and Methods

### 2.1. Basic Principle of PPM

**Figure 1:**
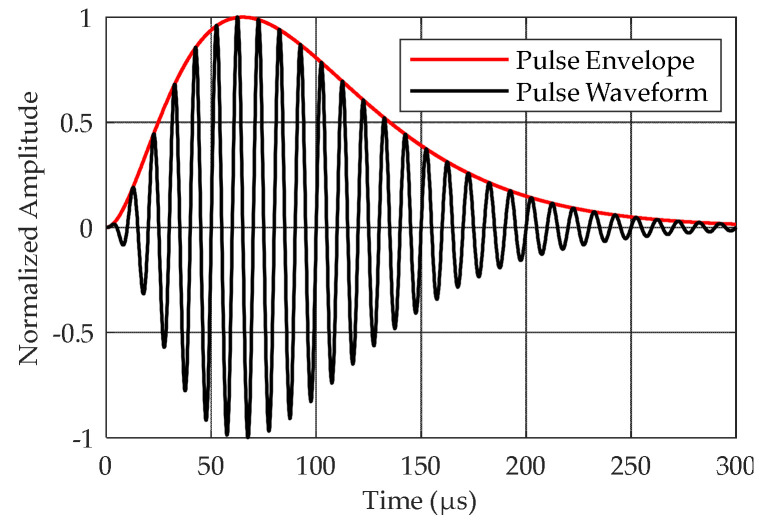
Reference pulse of Loran-C signal.

**Figure 2:**
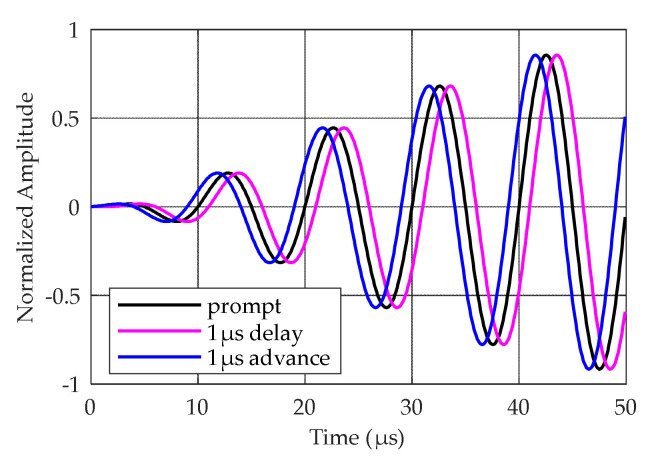
Pulse position modulation (PPM) signal.

The Loran-C signal has been formally defined by the United States Coast Guard (USCG) as a sequence of pulses in the radio frequency (RF) energy range with a central frequency of 100 kHz [31]. The definition of a single Loran-C pulse can be found in [31]. The first pulse in the Loran-C pulse group is called the reference pulse, as shown in Figure 1. The EUROFIX modulation scheme uses the last six pulses of the Loran-C pulse group. These pulses are pulse position modulated by ±1 μs (1 μs advance, a prompt, or 1 μs delay) [13], as shown in Figure 2. To minimize the impact to users, PPM encoding uses 128 of the possible 141 balanced patterns to represent seven bits of data per group repetition interval (GRI). In this article, pulses with modulated data in the Loran-C pulse group are called data pulses in short.

### 2.2. Envelope Model of Loran-C Pulse

Let *A*(*n*) denote the normalized envelope of the reference pulse, as shown in Figure 1, where n=0,1,⋯N−1 represents the sampling time. In this study, the sampling rate of the system is assumed to be 1 MHz (i.e., the sampling interval is 1 μs). The PPM time shifts are equivalent to a phase shift. Since a Loran-C signal has a period of 10 μs, a 1 μs advance is equivalent to a–π/5 radian shift, a 1 μs delay is equivalent to a π/5 radian shift, and no time shift is equivalent to a zero radian shift. Typically, the Loran-C receiver receives a mixed signal containing SWI, groundwave signal, and noise [19,20,21]. The skywave-to-groundwave amplitude ratio is represented by *λ*, and the delay of the SWI is represented by *τ*. Let m∈{1,2,3,4,⋯,8} denote one of the *m*-th Loran-C pulse envelopes in the Loran-C pulse group; thus, the mathematical model of the Loran-C pulse envelope can be expressed as:

(1) $$\begin{array}{ll} r_{m}(n) & =V_{op}B(n)e^{i(\xi_{m}+\varphi_{0})}+w_{m}(n)\\ \xi_{m} & =\theta_{m}+\phi_{m}\\ B(n) & =A(n)+\lambda A(n-\tau )e^{\tau \pi /5}\\ & =e^{i\psi_{n}}\sqrt{A^{2}(n)+2\lambda A(n)A(n-\tau )\cos(\tau \pi /5)+\lambda^{2}A(n-\tau )}\\ \psi_{n} & =\tan^{-1}\left(\frac{\lambda A(n-\tau )\sin(\tau \pi /5)}{A(n)+\lambda A(n-\tau )\cos(\tau \pi /5)}\right), \end{array}$$

where $V_{op}$ is the amplitude, B(n) is the Loran-C pulse envelope with the SWI, $w_{m}(n)$ is the white Gaussian noise with zero mean and variance $\sigma_{w}^{2}$, $\theta_{m}$ is the modulation phase ($\theta_{1}$ is equal to 0), $\phi_{m}$ is the phase code (0 or π, and $\phi_{1}$ is equal to 0), and $\varphi_{0}$ is the initial phase of the carrier.

To reduce the complexity of demodulation, and considering that the energy of a Loran-C pulse signal is mainly concentrated near the envelope peak, we take the duration of the Loran-C pulse signal as 200 μs, i.e., *N* is equal to 200. According to [20], *τ ≥* 35 μs. Therefore, the range of *τ* discussed in this article is 35–200 μs. In addition, to distinguish the data pulse from the reference pulse, the subscript *k* is used to express the data pulse, where k∈{3,4,⋯,8}.

### 2.3. PPM Demodulation Method

#### 2.3.1. Description of the PPM Demodulation Method

**Figure 3:**
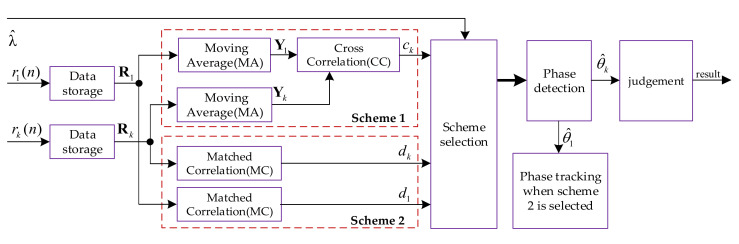
Flow diagram of the PPM demodulation method employed in this study.

In this study, the EC–PD method is used for a low SNR demodulation of the PPM, in which EC has two implementation schemes: MA–CC and MC. Before the demodulation, the receiver needs to complete the acquisition of the Loran-C pulse signal, which is used to determine the starting positions of the reference and data pulses. Moreover, it needs to identify the skywave to obtain the estimated values of λ Figure 3 shows the flow diagram of the demodulation method.

The first step in the demodulation process is to store the envelope sampling data (ESD) of the reference and data pulses; the storage depth is *N*. The stored ESD of the reference and data pulses are represented by column vectors $R_{1}$ and $R_{k}$, respectively, as follows:

(2) $$\begin{array}{l} R_{1}=e^{i\varphi_{0}}V_{op}B+W_{1}\\ R_{k}=e^{i(\xi_{k}+\varphi_{0})}V_{op}B+W_{k} \end{array}$$

where

$$\begin{array}{l} B={\left[B(0),B(1),\cdots ,B(N-1)\right]}^{T},\\ W_{1}={\left[w_{1}(0),w_{1}(1),\cdots ,w_{1}(N-1)\right]}^{T},\\ W_{k}={\left[w_{k}(0),w_{k}(1),\cdots ,w_{k}(N-1)\right]}^{T}. \end{array}$$

In scheme 1, the MA technique is first used to process the stored ESD of the reference and data pulses, and the results are recorded as $Y_{1}$ and $Y_{k}$, respectively. Subsequently, $c_{k}=Y_{1}^{H}Y_{k}$ is obtained through the CC between $Y_{1}$ and $Y_{k}$. In scheme 2, $d_{1}=A^{H}R_{1}$ and $d_{k}=A^{H}R_{k}$ are obtained through the MC, where $A={\left[A(0),A(1),\cdots ,A(N-1)\right]}^{T}$. The scheme selector makes a selection between schemes 1 and 2 based on $\hat{\lambda }$ (estimated value ofλ). When scheme 1 is selected, the selector outputs $c_{k}$; otherwise, it outputs $d_{1}$ and $d_{k}$. The scheme selection strategy is given in Section 2.3.4. In addition, if scheme 1 is selected, the phases introduced by the skywave and the carrier can be eliminated through the CC, and then a non-coherent demodulation is carried out. However, if scheme 2 is selected, a phase tracking loop is required to eliminate the phases introduced by the skywave and the carrier.

The phase detection can be carried out using an inverse tangent function with return values in the interval [−π/2,π/2] to eliminate the phase code given that it has only two values, 0 or π. We record the phase detection result as ${\hat{\theta }}_{k}$, which can be expressed as follows:

(3) $${\hat{\theta }}_{k}=\left\{\begin{array}{cc} \mathrm{atan}\left(\frac{\mathrm{imag}(c_{k})}{\mathrm{real}(c_{k})}\right) & \mathrm{when}\;\mathrm{scheme}\;1\;\mathrm{is}\;\mathrm{selected},\\ \mathrm{atan}\left(\frac{\mathrm{imag}(d_{k})}{\mathrm{real}(d_{k})}\right) & \mathrm{when}\;\mathrm{scheme}\;2\;\mathrm{is}\;\mathrm{selected}, \end{array}\right.$$

where atan(⋅) is the inverse tangent function, imag(⋅) and real(⋅) denote the imaginary and real parts of complex numbers, respectively. Additionally, then the demodulation judgment is made based on the following rules:

1) if $-\pi /10\leq {\hat{\theta }}_{k}\leq \pi /10$, the judgment is no time shift.
2) if ${\hat{\theta }}_{k}>\pi /10$, the judgment is 1 μs delay.
3) if ${\hat{\theta }}_{k}<-\pi /10$, the judgment is 1 μs advance.

Let G denote the SNR gain obtained by the EC, expressed as $G=SNR_{out}-SNR_{in}$, where $SNR_{out}$ is the selector output SNR, and $SNR_{in}$ is the RF input SNR. Furthermore, let $G_{sche1}$ and $G_{sche2}$ denote the SNR gains of schemes 1 and 2, respectively. Evidently, $G_{sche1}$ and $G_{sche2}$ are not equivalent. The SNR gain is one of the key parameters used to evaluate the performance of the EC–PD method. Therefore, the calculation and analysis of the SNR gain will be focused upon next.

#### 2.3.2. Mathematical Model of the EC

This section presents the mathematical models of the EC schemes, laying a foundation for the calculation and analysis of the SNR gain.

First, we define a N×N real symmetric moving average matrix, denoted by Q, which is determined by N and R, where R is the moving window radius of the MA. For example, when N=5 and R=2, Q can be expressed as follows:

(4) $$Q=\frac{1}{2R+1}\left[\begin{array}{ccccc} 1 & 1 & 1 & 0 & 0\\ 1 & 1 & 1 & 1 & 0\\ 1 & 1 & 1 & 1 & 1\\ 0 & 1 & 1 & 1 & 1\\ 0 & 0 & 1 & 1 & 1 \end{array}\right].$$

In other words, when the stored ESD are processed by the MA, it is equivalent to adding *R* zeros before and after the stored ESD, and then replacing the middle value of 2R+1 points with the average. Thus, $c_{k}$ in scheme 1 can be further expressed as:

(5) $$\begin{array}{ll} c_{k} & =Y_{1}^{H}Y_{k}={\left(QR_{1}\right)}^{H}QR_{k}\\ & =\left(e^{-i\varphi_{0}}V_{op}B^{H}Q^{H}+W_{1}^{H}Q^{H}\right)\times \left(e^{i(\xi_{k}+\varphi_{0})}V_{op}QB+QW_{k}\right)\\ & =e^{i\xi_{k}}V_{op}^{2}B^{H}Q^{2}B+I_{k}, \end{array}$$

where $I_{k}$ is the noise introduced by the CC and can be expanded as follows:

(6) $$I_{k}=e^{-i\varphi_{0}}V_{op}B^{H}Q^{2}W_{k}+e^{i(\xi_{k}+\varphi_{0})}V_{op}W_{1}^{H}Q^{2}B+W_{1}^{H}Q^{2}W_{k}.$$

Evidently, $B^{H}Q^{2}B$ in Equation (5) does not contain any phase information. Therefore, scheme 1 can realize non-coherent demodulation without an additional carrier phase tracking loop.

In scheme 2, $d_{1}=A^{H}R_{1}$, and using Equation (2), we can rewrite $d_{1}$ as

(7) $$d_{1}=e^{i\varphi_{0}}V_{op}A^{H}B+A^{H}W_{1}=e^{i[\varphi_{0}+f_{ph}(\lambda ,\tau )]}V_{op}f_{am}(\lambda ,\tau )+A^{H}W_{1},$$

where $f_{ph}(\lambda ,\tau )$ and $f_{am}(\lambda ,\tau )$ represent the phase and amplitude of $A^{H}B$, respectively. According to Equation (7), when there is a skywave interference in the received signal, the MC will introduce an interference phase, which affects the detection of the modulation phase. Therefore, in the demodulation process of scheme 2, it is necessary to use the carrier phase tracking loop to ensure that $\varphi_{0}+f_{ph}(\lambda ,\tau )$ approaches zero. In this study, we assume $\varphi_{0}+f_{ph}(\lambda ,\tau )=0$; thus, $d_{k}$ can be expressed as:

(8) $$\begin{array}{ll} d_{k} & =A^{H}R_{k}=e^{i(\xi_{k}+\varphi_{0})}V_{op}A^{H}B+A^{H}W_{k}\\ & =e^{i[\xi_{k}+\varphi_{0}+f_{ph}(\lambda ,\tau )]}V_{op}f_{am}(\lambda ,\tau )+A^{H}W_{k}\\ & =e^{i\xi_{k}}V_{op}f_{am}(\lambda ,\tau )+A^{H}W_{k}. \end{array}$$

#### 2.3.3. SNR Gain

From Equation (6), we can easily prove that the noise terms in $I_{k}$ are independent of each other. Therefore, the mean of $I_{k}$ is equal to zero, and its variance $\sigma^{2}$ can be given as:

(9) $$\begin{array}{ll} \sigma^{2} & =E\{I_{k}I_{k}^{H}\}\\ & =V_{op}^{2}E\left\{B^{H}Q^{2}W_{k}W_{k}^{H}Q^{2}B\right\}+V_{op}^{2}E\left\{W_{1}^{H}Q^{2}BB^{H}Q^{2}W_{1}\right\}+E\left\{W_{1}^{H}Q^{2}W_{k}W_{k}^{H}Q^{2}W_{1}\right\}\\ & =2V_{op}^{2}\sigma_{w}^{2}B^{H}Q^{4}B+q\sigma_{w}^{2}\sigma_{w}^{2}, \end{array}$$

where q is the trace of matrix $Q^{4}$. According to Equations (5) and (9), the output SNR of scheme 1 can be expressed as:

(10) $$\begin{array}{ll} SNR_{out} & =10\log_{10}\left(\frac{V_{op}^{4}\alpha^{2}}{\sigma^{2}}\right)\\ & =10\log_{10}\left(\frac{\alpha^{2}}{2\beta +q\sigma_{w}^{2}/V_{op}^{2}}\right)+10\log_{10}\frac{V_{op}^{2}}{\sigma_{w}^{2}}, \end{array}$$

where $\alpha =B^{H}Q^{2}B$, and $\beta =B^{H}Q^{4}B$. Since $SNR_{in}=10\log_{10}(V_{op}^{2}/\sigma_{w}^{2})$, the equation for calculating the SNR gain of scheme 1 can be derived as follows:

(11) $$\begin{array}{ll} G_{sche1} & =SNR_{out}-SNR_{in}\\ & =10\log_{10}\left(\frac{\alpha^{2}}{2\beta +q\times 10^{-SNR_{in}(dB)/10}}\right). \end{array}$$

**Figure 4:**
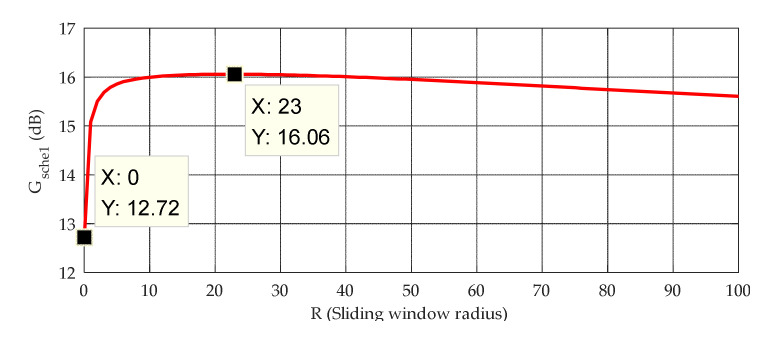
Relationship between $G_{sche1}$ and R in the case of $SNR_{in}=0\;\mathrm{dB}$.

The above equation indicates that in the case of low SNR, the SNR gain of scheme 1 can be increased by reducing q. A simulation is carried out to determine the value of *R*. In this simulation, we set $SNR_{in}=0\;\mathrm{dB}$. Figure 4 shows the result. As shown, when R=0 (i.e., the MA is not adopted), $G_{sche1}$ is only 12.72 dB, whereas when R=23, $G_{sche1}$ reaches the maximum value of 16.06 dB. When R≥23, q is small enough, so that the effect of $SNR_{in}$ on $G_{sche1}$ can be ignored. However, with further increase in R, the loss in the signal energy due to the MA is evident, thereby reducing $G_{sche1}$. To sum up, we set the sliding window radius of the MA as 23.

According to Equation (7), and from the above analysis process, we can easily obtain the equation for calculating the SNR gain of scheme 2, as follows:

(12) $$G_{sche2}=10\log_{10}\left(\frac{{\left|f_{am}(\lambda ,\tau )\right|}^{2}}{A^{H}A}\right).$$

#### 2.3.4. Selection of EC Schemes

**Figure 5:**
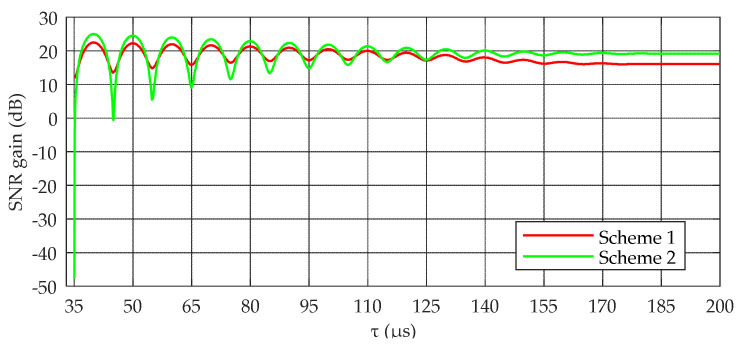
Relationship between signal-to-noise ratio (SNR) gain and τ when λ=1.6 dB.

The magnitude of the SNR gain is an important basis for selecting the EC schemes. Evidently, when there is no SWI, $G_{sche2}$ is at least 3 dB higher than $G_{sche1}$. Thus, scheme 2 is the best scheme under a Gaussian channel. However, in the presence of SWI, the selection of the EC schemes is more complicated. In Figure 5, the relationship between the SNR gain and the delay of the SWI is simulated in the case of λ=1.6 dB. The SNR gain fluctuates with the change in the delay. This is mainly because when the delay is an odd multiple of 5 μs, the coincidence part of the skywave and the groundwave will cancel each other, and the SNR gain will be reduced. On the contrary, when the delay is an even multiple of 5 μs, the coincidence part of the skywave and the groundwave will overlap each other, which is conducive to the SNR gain. In addition, when the coincidence part of the skywave and the groundwave cancel each other, A(n) and B(n) will be seriously mismatched, which will lead to a sharp deterioration in the SNR gain of scheme 2, whereas scheme 1 will not cause mismatching owing to its CC. Therefore, in the extreme case, the deterioration degree of the SNR gain of scheme 1 is significantly less than that of scheme 2. In other words, the robustness of scheme 1 under a CWI environment is significantly better than that of scheme 2.

There are two strategies, namely strategy A and strategy B, for selecting the EC schemes. In strategy A, the receiver needs to obtain $\hat{\lambda }$ and $\hat{\tau }$ through skywave identification, calculate $G_{sche1}$ and $G_{sche2}$ using Equations (11) and (12), respectively, and finally select the EC scheme with a high SNR gain. In strategy B, the receiver compares $\hat{\lambda }$ with the threshold value $\lambda_{thred}$; when $\hat{\lambda }\geq \lambda_{thred}$, scheme 1 is selected; otherwise, scheme 2 is selected. In strategy A, the receiver is required to estimate λ and τ with a very high accuracy; otherwise, it will cause a large deviation between the calculation result of the SNR gain and the real value, thus making the scheme selection invalid. In strategy B, the receiver only needs to estimate λ, which is relatively simple to implement. Therefore, this strategy is recommended for selecting the EC schemes in this study.

**Figure 6:**
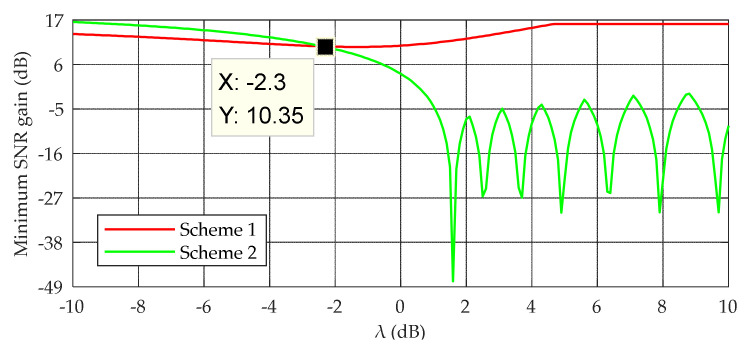
Minimum SNR gain as a function of λ.

We present a method to determine $\lambda_{thred}$ based on the minimum SNR gain, where the minimum SNR gain refers to the minimum value that the SNR gain can reach when the skywave-to-groundwave amplitude ratio is λ. According to Equations (11) and (12), the minimum SNR gain of the two schemes can be obtained by simulation, as shown in Figure 6. The simulation results show that when λ≥−2.3   dB, the minimum SNR of scheme 1 is greater than that of scheme 2; otherwise, the minimum SNR of scheme 1 is less than that of scheme 2. Therefore, $\lambda_{thred}$ is set to −2.3 dB in this study. This method can overcome the problem where the SNR ratio gain deteriorates rapidly in extreme cases, thereby improving the robustness and stability of anti-SWI demodulation.

## 3. Results

### 3.1. Validation Method

We verified the effectiveness of the demodulation method from three aspects. In Section 3.2, the simulation results of the error probability under Gaussian channel for the EC–PD, EPD–MD, and basic methods are given. In Section 3.3, we consider the influence of the SWI on the demodulation to further analyze and compare the demodulation performances of the EC–PD and EPD–MD methods. In Section 3.4, an experimental verification platform set up to receive and demodulate an actual Loran-C signal is presented, and the demodulation performance of the EC–PD method is verified.

### 3.2. Anti-Noise Performance

**Figure 7:**
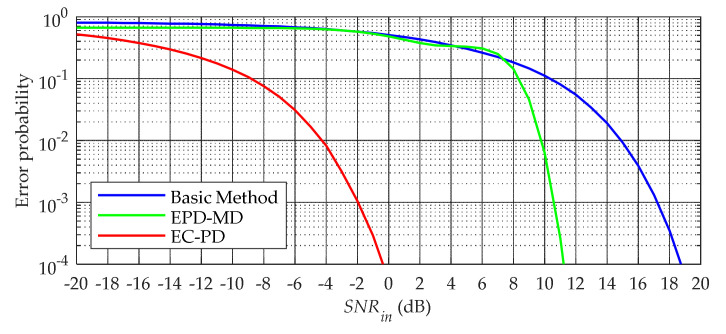
Probability of error as a function of $SNR_{in}$.

Figure 7 shows the simulation results of the error probability under Gaussian channel. When the $SNR_{in}$ is greater than 8 dB, the demodulation performance of the EPD–MD method is better than that of the basic method, thus demonstrating the effectiveness of the EPD–MD method at high SNRs. However, with the decrease in the $SNR_{in}$, its demodulation performance deteriorates sharply. The demodulation performance of the EC–PD method is significantly better than that of the EPD–MD and basic methods. For example, when the $SNR_{in}$ is in the range of −9–0 dB, the error probability of the EC–PD method is lower than that of the EPD–MD method by one to four orders of magnitude.

In addition, we take the SNR threshold as one of the quality parameters to compare the demodulation performances of the above three methods. The SNR threshold mentioned in this article refers to the minimum $SNR_{in}$ required to make the error probability no more than 10^−3^, and is recorded as $SNR_{th}$ The simulation results show that the $SNR_{th}$ of the EC–PD method is only −2 dB, which is 12.5 and 19.5 dB lower than that of the EPD–MD and basic methods, respectively.

### 3.3. Anti-SWI Performance

**Figure 8:**
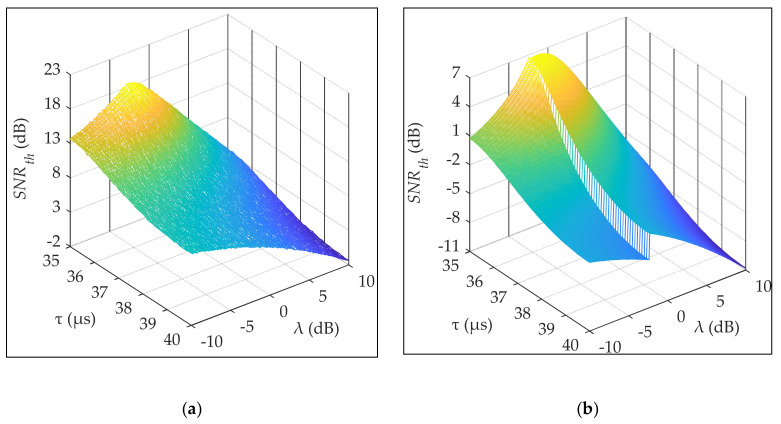
Demodulated $SNR_{th}$ as a function of τ and λ: (**a**) envelope phase detection–majority decision (EPD–MD) method; (**b**) envelope correlation–phase detection (EC–PD) method.

In this section, the $SNR_{th}$ is used to simulate and compare the anti-SWI performances of the EC–PD and EPD–MD methods. As demonstrated in Section 2.3.4, the SWI is most unfavorable when λ = 35 μs and most favorable when λ = 40 μs, for demodulation. Therefore, in the simulation, λ is assigned a range of 35–40 μs with a resolution of 0.1 μs. Moreover, the $SNR_{in}$ is assigned a range of −10–10 dB with a resolution of 0.1 dB. Figure 8 shows the simulation results. In Figure 8, it can be observed that: (1) The range of $SNR_{th}$ required for the EPD–MD method is −2–19 dB, and the dynamic value is 21 dB; (2) The range of $SNR_{th}$ required for the EC–PD method is −11–7 dB, and the dynamic value is 18 dB. Compared with the EPD–MD method, the robustness (represented by the maximum $SNR_{th}$) of the anti-SWI demodulation of the EC–PD method is improved by 14 dB, and the stability (represented by the dynamic value) is improved by 3 dB.

**Figure 9:**
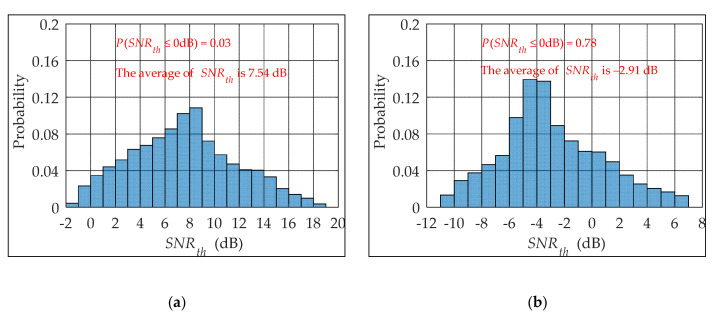
Statistical histograms of $SNR_{th}$: (**a**) EPD–MD method; (**b**) EC–PD method.

Furthermore, based on the above simulation data, we determined the statistical characteristics of the SNR threshold, represented by the probability distribution, as shown in Figure 9. In Figure 9, it can be observed that: (1) The values of the probability of $SNR_{th}$ are less than 0 for the EPD–MD and EC–PD methods are 0.03 and 0.78, respectively; (2) The average $SNR_{th}$ values required for the EPD–MD and EC–PD methods are 7.54 and −2.91 dB, respectively. The EC–PD method can still achieve data demodulation at low SNRs under the SWI environment.

### 3.4. Experimental Verification

**Figure 10:**
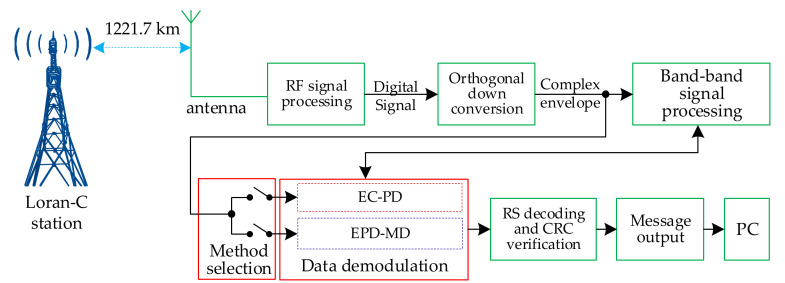
Test platform of data demodulation method.

We test the data demodulation method with signals transmitted by a real Loran-C system. The actual received signal is the Loran-C signal (the GRI is 74.30 ms) emitted by the main station (station ID is 09) of Shandong Rongcheng. This station belongs to China’s Changhe 2 navigation system. The test platform is placed in national time service center of China, 1227.1 km away from the station. Figure 10 shows the test platform.

**Figure 11:**
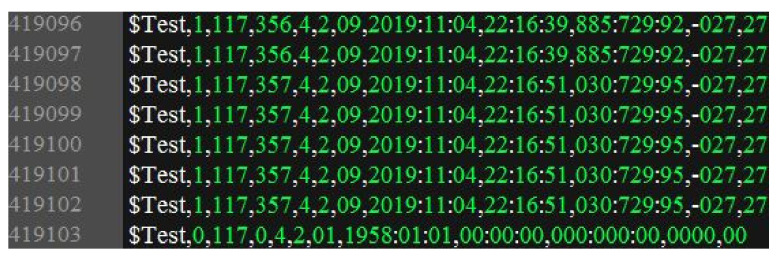
Screenshot of some experimental data.

The following is the description of the test platform:

(1). In RF signal processing, the input Loran-C signal is sampled by analogue-to-digital and filtered by an adaptive notch and finite impulse response band-pass, thus obtaining the digital signal.
(2). The complex envelope of the Loran-C pulse is obtained through orthogonal down conversion.
(3). The baseband signal processing includes signal acquisition [16], carrier phase tracking, and skywave identification [20]. The signal acquisition step provides the starting positions of the reference and data pulses, and the skywave identification provides the estimated value of λ.
(4). The EC–PD and EPD–MD methods are alternately selected for signal demodulation every half an hour.
(5). The experimental data are composed of message frames, as shown in Figure 11. The serial port outputs one message frame to the PC every second, including $test ID, method ID (“0” references the EPD–MD method, and “1” refers to the EC–PD method), experimental period, number of correct message frames in each experimental period, message type, message subtype, station ID, time code 1 (yyyy:mm:dd), time code 2 (hh:mm:ss), precise time information (ms:μs:10ns), broadcasting deviation, and leap second. The correctness of the message frame is examined by Reed-Solomon (RS) decoding and cyclic redundancy check (CRC).

**Figure 12:**
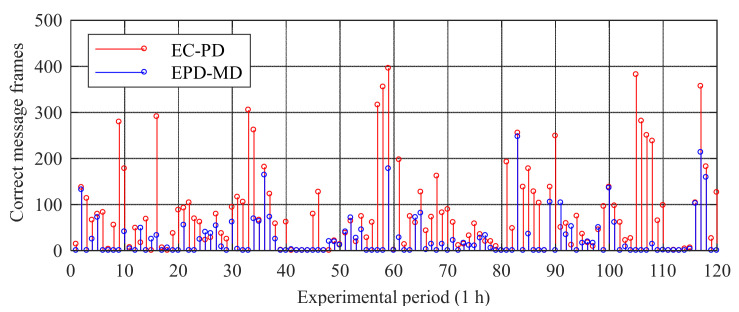
Statistical results of experimental data.

Figure 12 shows the number of correct message frames of the two demodulation methods in each experimental period. As shown, the demodulation performance of the EPD–MD method is similar to that of the EC–PD method in only a few experimental periods, whereas in most experimental periods, the demodulation performance of the EC–PD method is significantly better than that of the EPD–MD method.

In the next step, we define two variables, namely the maximum data availability $\eta_{\max}$ and average data availability $\eta_{\mathrm{avg}}$, to compare the effectiveness of the two demodulation methods in detail, as follows

(13) $$\eta_{\max}=F_{t}\times \frac{2CMF_{max}}{EP}\times 100\;,\;\eta_{avg}=F_{t}\times \frac{2CMF_{all}}{EP\times NP}\times 100,$$

where $CMF_{max}$ is the maximum number of correct message frames in a certain experimental period, EP=3600 s is the duration of one experimental period, $F_{t}=30\times GRI$ is the duration of one message frame, $CMF_{all}$ is the total number of correct message frames during the entire experimental time, and NP=120 is the total number of experimental periods. Since the GRI of the received Loran-C signal is 74.30 ms, we have $F_{t}=2.229\;s$. From the experimental data: (1) The $CMF_{max}$ values of the EC–PD and EPD–MD methods are 396 and 247, respectively, and their $\eta_{\max}$ values are calculated to be 49 and 30.6% respectively; (2) The $CMF_{all}$ values of the EC–PD and EPD–MD methods are 10526 and 3190, respectively, obtained by summing up the number of correct message frames in each experimental period; the $\eta_{avg}$ values of the EC–PD and EPD–MD methods are 0.9 and 3.3%, respectively. The calculation results show that compared with the EPD–MD method, the $\eta_{\max}$ and $\eta_{avg}$ values of the EC–PD method are increased by approximately 1.6 and 3.3 times, respectively.

## 4. Discussion

The realization and application of the Loran-C system data link technology can make it possible to build a relatively perfect PNT system by combining with the satellite-based PNT system. However, the existing demodulation method used in the Loran-C system cannot effectively suppress noise and SWI. Therefore, with the development of modern Loran-C systems, a more advanced Loran-C signal processing capability is required. In this study, we developed a Loran-C demodulation method at low SNRs based on the EC–PD, where EC includes two schemes: MA–CC and MC. The mathematical models of the MA–CC and MC, calculation of the SNR gain, and selection of the EC schemes based on the skywave identification results were described in detail. The theoretical analysis results showed that the MA–CC is more suitable for scenarios with SWI, whereas the MC is more suitable for scenarios with only noise. Therefore, the combination of the MA–CC and MC could effectively reduce the effects of noise and SWI on the demodulation process.

In addition, a simulation was conducted to verify the effectiveness of the demodulation method and analyze its anti-noise and anti-SWI performances. The simulation results showed that compared with the existing method in [14], the proposed method has clear advantages: (1) The demodulation SNR threshold under Gaussian channel is only −2 dB, which represents a reduction of 12.5 dB by comparison; (2) The probability of the demodulated SNR threshold being less than zero under the SWI environment can reach 0.78, which is a 26-fold increase by comparison, and the robustness and stability of anti-SWI demodulation are improved by 14 and 3 dB, respectively. Finally, we set up an experimental verification platform that can receive and demodulate an actual Loran-C signal. The test results showed that the average data availability of our demodulation method is 3.3 times higher than that of the method proposed in [14]. Thus, our demodulation method has a higher engineering application value, and has been optimized for the design of new Loran-C timing receivers. This will improve the performance of modern Loran-C systems, making them a more reliable backup for the GNSS.

The EC technology proposed in this article has a very low implementation complexity compared with some techniques, such as singular value decomposition [32,33] and wavelet transform [34,35], which involve a lot of complex multiplication operations to improve the SNR. The combination of EC and the above technologies could be an effective way to further improve the Loran-C data demodulation performance in the future.

## References

[1] Yang Y.X. (2016). Concepts of Comprehensive PNT and Related Key Technologies. Acta Geod. Cartogr. Sin..

[2] Yang S.H., Lee C.B., Lee Y.K., Lee J.K. (2011). Accuracy Improvement Technique for Timing Application of LORAN-C Signal. IEEE Trans. Instrum. Meas..

[3] Lo S.C., Peterson B.B., Enge P.K., Swaszek P. (2007). Loran Data Modulation: Extensions and Examples. IEEE Trans. Aerosp. Electron. Syst..

[4] Wang X.Y., Zhang S.F., Sun X.W. (2017). The Additional Secondary Phase Correction System for AIS Signals. Sensors.

[5] Qiu D., Boneh D., Lo S.C., Enge P.K. (2010). Reliable Location-Based Services from Radio Navigation Systems. Sensors.

[6] Griffioen J.W., Oonincx P.J. (2013). Suitability of Low-Frequency Navigation Systems for Artillery Positioning in a GNSS Denied Environment. J. Navig..

[7] Liu Y.H., Li X.H., Liu C.H., Li S.F. (2017). Research on the integrated positioning techniques of ground-based LF time service system and GNSS. J. Time Freq..

[8] Kim H., Lee J., Oh S.H., So H., Hwang D.H. (2019). Multi-Radio Integrated Navigation System M&S Software Design for GNSS Backup under Navigation Warfare. Electronics.

[9] Son P.W., Park S.H., Seo K., Han Y., Seo J. Development of the Korean eLoran testbed and analysis of its expected positioning accuracy. Proceedings of the 19th IALA Conference.

[10] Lo S.C., Peterson B.B., Hardy T., Enge P.K. (2010). Improving Loran coverage with low power transmitters. J. Navig..

[11] EU eLoran Efforts Sharpen While U.S. Requirements Study Continues. https://insidegnss.com/eu-eloran-efforts-sharpen-while-u-s-requirements-study-continues/.

[12] Offermans G., Bartlett S., Schue C. (2017). Providing a Resilient Timing and UTC Service Using eLoran in the United States: Resilient timing using eLoran. Navigation.

[13] Willigen D.V., Offermans G.W.A., Helwig A.W.S. EUROFIX: Definition and current status. Proceedings of the IEEE Position Location & Navigation Symposium.

[14] Li S.F., Wang Y.L., Hua Y., Xu Y.L. (2012). Research of Loran-C data demodulation and decoding technology. Chin. J. Sci. Instrum..

[15] Li Y., Hua Y., Yan B.R., Guo W. (2019). Analysis on Time Variation Analysis of BPL Long Wave Time Service Signal Transmission Delay. J. Astronaut. Metrol. Meas..

[16] Li S.F., Wang Y.L., Hua Y., Yuan J.B. (2013). Loran-C Signal Fast Acquisition Method and Its performance Analysis. J. Electron. Inf. Technol..

[17] Yan W.H., Zhao K.J., Li S.F., Wang X.H., Hua Y. (2020). Precise Loran-C Signal Acquisition Based on Envelope Delay Correlation Method. Sensors.

[18] Gao Y.Y., Hua Y., Li S.F., Yang C.Z. Acquisition method of Loran-C signal based on matched filter. Proceedings of the 2015 IEEE International Conference on Signal Processing, Communications and Computing.

[19] Zhang K., Wan G.B., Li M.C., Xi X.L. (2019). Skywave delay estimation in Enhanced Loran based on extended invariance principle weighted Fourier transform and relaxation algorithm. IET Radar Sonar Navig..

[20] Wu H.R., Liu R.Z. (2009). A new Algorithm for Sky-Wave and Ground-Wave Detection of Loran C Based on FFT/IFFT Technology. J. Nav. Aeronaut. Astronaut. Univ..

[21] Zhang K., Wan G.B., Xi X.L. (2019). Enhanced Loran skywave delay estimation based on artificial neural network in low SNR environment. IET Radar Sonar Navig..

[22] Zhang K., Wan G.B., Pu Y., Zheng C., Xi X.L. (2017). Loran-C skywave delay estimation using hybrid-WRELAX algorithm. Electron. Lett..

[23] Wu M., Li F.N., Su X.Q., Wang G.C. (2013). The New Method of Loran C Cycle Identification Based on Gaussian Smoothing Filter. Hydrogr. Surv. Charting.

[24] Yan W.H., Hua Y., Yuan Y.B., Zhao K.J., Li S.F. A joint detection method of cycle-identification for loran-C signal. Proceedings of the 2017 IEEE International Conference on Electronic Measurement & Instruments.

[25] Tehrani A.K.Z., Pourmohammad A. Acurate and Robust Loran-C Cycle Identification. Proceedings of the 2017 IEEE International Conference on Application of Information and Communication Technologies.

[26] Li Y., Hua Y., Yan B.R., Guo W. (2019). Experimental Study on a Modified Method for Propagation Delay of Long Wave Signal. IEEE Antennas Wirel. Propag. Lett..

[27] Wang D.D., Xi X.L., Pu Y.R., Liu J.F., Zhou L.L. (2016). Parabolic Equation Method for Loran-C ASF Prediction Over Irregular Terrain. IEEE Antennas Wirel. Propag. Lett..

[28] Son P.W., Rhee J.H., Hwang J., Seo J. (2019). Universal Kriging for Loran ASF Map Generation. IEEE Trans. Aerosp. Electron. Syst..

[29] Lo S.C., Peterson B.B., Enge P.K. (2007). Loran Data Modulation: A Primer[AESS Tutorial IV]. IEEE Aerosp. Electron. Syst. Mag..

[30] Lo S.C., Enge P.K. Data transmission using LORAN-C. Proceedings of the International Loran Association 29th Annual Meeting.

[31] U.S Coast Guard and the U.S Coast Guard Auxiliary Loran-C User Handbook. https://www.loran.org/otherarchives/-1992%20-Loran-C%20User%20Handbook%20-%20USCG.pdf.

[32] Zha X., Ni S.H., Zhang P. (2015). Effective Iteration Method of a Class of Nonlinear Signal Denoising Based on Singular Value Decomposition. J. Electron. Inf. Technol..

[33] Guo Q., Zhang C., Zhang Y., Liu H. (2016). An Efficient SVD-Based Method for Image Denoising. IEEE Trans. Circuits Syst. Video Technol..

[34] Lilly J.M., Olhede S.C. (2010). On the Analytic Wavelet Transform. IEEE Trans. Inf. Theory.

[35] Srivastava M., Anderson C.L., Freed J.H. (2016). A New Wavelet Denoising Method for Selecting Decomposition Levels and Noise Thresholds. IEEE Access.

